# Comparative Analysis for Glycopatterns and Complex-Type *N-*Glycans of Glycoprotein in Sera from Chronic Hepatitis B- and C-Infected Patients

**DOI:** 10.3389/fphys.2017.00596

**Published:** 2017-08-21

**Authors:** Xinmin Qin, Yonghong Guo, Haoqi Du, Yaogang Zhong, Jiaxu Zhang, Xuetian Li, Hanjie Yu, Zhiwei Zhang, Zhansheng Jia, Zheng Li

**Affiliations:** ^1^Laboratory for Functional Glycomics, College of Life Sciences, Northwest University Xi'an, China; ^2^Department of Infectious Diseases, Second Affiliated Hospital of Xi'an Jiaotong University Xi'an, China; ^3^Center of Infectious Diseases, Tangdu Hospital, Fourth Military Medical University Xi'an, China

**Keywords:** chronic hepatitis B, chronic hepatitis C, serum, glycoprotein, *N-*glycan, glycopatterns

## Abstract

**Background:** Chronic infection with HBV (CHB) or HCV (CHC) is the most common chronic viral hepatitis that can lead to cirrhosis and hepatocellular carcinoma in humans, their infections have distinct pathogenic processes, however, little is known about the difference of glycoprotein glycopatterns in serum between hepatitis B virus (HBV)- and hepatitis C virus (HCV)-infected patients.

**Methods:** A method combining the lectin microarrays, letin-mediated affinity capture glycoproteins, and MALDI-TOF/TOF-MS was employed to analyze serum protein glycopatterns and identify the glycan structures from patients with CHB (*n* = 54) or CHC(*n* = 47), and healthy volunteers (HV, *n* = 35). Lectin blotting was further utilized to validate and assess the expression levels of their serum glycopatterns. Finally, the differences of the glycoprotein glycopatterns were systematically compared between CHB and CHC patients.

**Conclusions:** As a result, there were 11 lectins (e.g., HHL, GSL-II, and EEL) exhibited significantly increased expression levels, and three lectins (LCA, VVA, and ACA) exhibited significantly decreased expression levels of serum protein glycopatterns only in the CHB patients. However, DBA exhibited significantly decreased expression levels, and two lectins (WGA and SNA) exhibited significantly increased expression levels of serum glycopatterns only in the CHC patients. Furthermore, LEL and MAL-I showed a coincidentally increasing trend in both CHC and CHB patients compared with the HV. The individual analysis demonstrated that eight lectins (MPL, GSL-I, PTL-II, UEA-I, WGA, LEL, VVA, and MAL-I) exhibited a high degree of consistency with the pooled serum samples of HV, CHB, and CHC patients. Besides, a complex-type *N-*glycans binder PHA-E+L exhibited significantly decreased NFIs in the CHB compared with HV and CHC subjects (*p* < 0.01). The MALDI-TOF/TOF-MS results of *N*-linked glycans from the serum glycoproteins isolated by PHA-E+L-magnetic particle conjugates showed that there was an overlap of 23 *N*-glycan peaks (e.g., m/z 1419.743, 1663.734, and 1743.581) between CHB, and CHC patients, 5 glycan peaks (e.g., m/z 1850.878, 1866.661, and 2037.750) were presented in virus-infected hepatitis patients compared with HV, 3 glycan peaks (1460.659, 2069.740, and 2174.772) were observed only in CHC patients. Our data provide useful information to find new biomarkers for distinguishing CHB and CHC patients based on the precision alteration of their serum glycopatterns.

## Introduction

Chronic infections with hepatitis B virus (HBV) or hepatitis C virus (HCV) are the most common chronic viral hepatitis in human. Some acute HBV infection can also turn into chronic HVB carriers, it is estimated that the number of chronic hepatitis B (CHB) patients worldwide is 350 million (Alter, 2003). It is generally accepted that at least 50% of the HBV carriers acquired their infections either prenatally or in early childhood (Jonas, 2009). Most of the acute HCV-infected patients turn into chronic carriers after 6 months, which is chronic hepatitis C (CHC). In despite of their distinct pathogenic processes of infections, both CHB and CHC can lead to cirrhosis, hepatic failure, and hepatocellular carcinoma (Arzumanyan et al., 2013). HBV serologic markers such as hepatitis B surface antigen (HBsAg), hepatitis B envelope antigen (HBeAg), hepatitis B core antigen (HBcAg), and anti-HCV antibody have been used in diagnosing and monitoring the progress of viral liver disease, some even has been introduced to evaluate treatment response to interferon (Sali et al., 2015). However, the dynamics of viral proteins expression and productions of the antibodies may vary, for instance, negative HBsAg cannot exclude HBV infection (He et al., 2003). Up to now, a definite diagnosis of HBV or HCV infected liver inflammation still relies on a combination of serological, biochemical, and histological examination (Chan, 2002).

Previous studies reported some serum/plasma microRNAs may serve as novel biomarkers for hepatitis or hepatocellular carcinoma such as miR-375, miR-125b-5p, and miR-1231 (Li et al., 2010; Giray et al., 2014; Hayes and Chayama, 2016). However, as a high potential family of biomarker for diseases, the glycan-related biomarker for hepatitis still remains unknown. With the development of bioinformatics and mass spectrometry-based techniques, the detection and analysis using mass spectrometry became a major method in the aspect of both laboratorial and clinical research against many diseases such as cancer (Jimenez and Verheul, 2014), hepatitis (Zhang et al., 2017), and congenital disorders (Xia et al., 2013). Generally speaking, using mass spectrometry-based techniques to study clinical samples or cell lines include several steps, after the preparation of the sample, the whole glycan can be derivatized, or tagged, as for specific glycan, it can be isolated by antibody or lectin. After the digestion of PNGase or trypsin, the target glycan can be subjected to LC MS/MS or MALDI-TOF/TOF MS to analyze quantitatively or qualitatively. This series of methods showed high throughput and efficiency, and has already been used by many researchers.

Glycosylation is a common post-translational modification of many secreted proteins and it is estimated that more than 70% of human proteins are glycosylated (Kuno et al., 2005). Glycosylation changes in serum proteins are highly associated with the development of liver disease. Serum glycan profiling is an early indicator for liver hepatocytes damage, which can provide critical diagnostic markers and insights into disease progression and pathogenesis (Dwek et al., 2002; Liu et al., 2007; Blomme et al., 2009). In response to inflammation stimuli, the serum levels of the acute phase proteins can increase to 1,000-fold, which are mostly glycoproteins. It has been demonstrated that *N-*linked glycan structures attached to these acute phase proteins also occur to change, such as the increased SLeX epitopes of haptoglobin, α1-acid glycoprotein (AGP), and α1-antichymotrypsin (Arnold et al., 2008). HBV is a DNA virus that integrates into the host genome. It is reported that HBV proteins have transcriptional transactivator activity, and the HBV x protein may has a strong *N-*acetylglucosaminyltransferase III (GnT-III) promotor-enhancing activity. Moreover, the increased accumulation of aberrant glycosylated apolipoprotein B (increased levels of bisecting GlcNAc) in intracelluar leads to inhibition of secretion of apolipoprotein B (Kang et al., 2004). In contrast, HCV is a positive stranded RNA virus which does not integrate into the host genome and it replicates in the cytoplasm. Some reports claimed that HCV proteins localize to the nucleus or interact with nuclear proteins, but it seems that viral proteins have more significant roles in hepatocarcinogenesis (Honda et al., 2006; Tsai and Chung, 2010). In chronic hepatitis caused by HBV and HCV-infected cases, glycosylation changes have been reported for several proteins isolated from the sera of patients, including IgG, haptoglobin, AGP, α2-macroglobin, transferrin, α-foetoprotein, caeruloplasmin, and α1-antitrypsin, these results have the potential as a new diagnosis biomarker and/or new therapies (Gornik and Lauc, 2008). Nevertheless, the difference between CHB and CHC in terms of glycoprotein glycopatterns in serum still remains unclear.

In this study, a method combining the lectin microarrays, lectin-mediated affinity capturing glycoproteins, and MALDI-TOF/TOF-MS was employed to analyze protein glycopatterns and identify the glycan profiles in sera from patients with CHB or CHC, and the differences between HBV and HCV-infected patients were systematically compared. The purpose of the present study is to reveal HBV/HCV-associated specific serum glycopatterns and glycan profiles, which may provide useful information to find the potential biomarkers for distinguishing of CHB and CHC patients based on the precise alterations of serum protein glycopatterns and anti-HBV/HCV therapeutic strategies.

## Materials and methods

### Study approval

The collection and use of all human pathological specimens for the research were approved by the Ethical Committee of Northwest University (Xi'an, China), the Ethical Committee of Second Affiliated Hospital of Xi'an Jiaotong University (Xi'an, China), and the Ethical Committee of Tangdu Hospital of Fourth Military Medical University (Xi'an, China). Written informed consent was received from participants for the collection of their whole serum. This study was conducted in accordance with the ethical guidelines of the Declaration of Helsinki.

### Serum collection

Serum samples were collected from 54 CHB, 47 CHC patients, and 35 healthy volunteers (HV). The diagnosis was based on clinical examination and biochemical tests. Liver function and hematological tests were performed using standard methods in a clinical setting. Hepatitis B markers were tested using a commercial radioimmunoassay (Kechuang, Shenzhen, China). HBV DNA levels were quantified using a real-time polymerase chain reaction assay (Qiagen, Shenzhen, China). HBV and HCV infected patients were tested for HBsAg and anti-HCV antibody respectively for more than 6 months. Exclusion criteria were coinfection with HCV or HBV and main complications of liver disease such as alcoholic hepatitis, drug-related hepatitis, and obstructive jaundice. Patients with CHB were positive for hepatitis B surface antigen and presented serum alanine aminotransferase (ALT) levels 2–10 times the upper limits of normal (ULN). Liver fibrosis stages were determined by using non-invasive transient elastography and serological marker-based algorithm. Patients were enrolled in the study performed mild fibrosis (F0–F1) and the detailed information of patients is described in Table 1.

**Table 1:** Characteristics of health volunteers and the patients of CHB and CHC.

**Parameter**	**HV**	**CHB**	**CHC**
Number of patients(*n*)	35	54	47
Gender, male/female	17/18	27/27	23/24
Age (years)	44 ± 12.4	46 ± 11.5	45 ± 13.7
HBsAg (%)	**–**	21.45	**–**
HBsAb (%)	**–**	12.68	**–**
HBeAg (%)	**–**	9.45	**–**
AST (IU/L)	**–**	51.39 ± 14.0	48.1 ± 12.0
ALT (IU/L)	20	94.1 ± 11.0	93.9 ± 15.0
AFP (ng/mL)	**–**	71.9 ± 7.9	81.0 ± 21.1
Albumin (g/dl)	**–**	40.0 ± 8.0	37.9 ± 14.0
Bilirubin (g/dl)	**–**	43.2 ± 6.8	56.8 ± 9.0
Total serum protein (g/L)	**–**	63.7 ± 7.0	68.0 ± 6.1
Liver fibrosis (1/2/3/4/unknown)	**–**	31/23/0/0/0	27/23/0/0/0

*AST, aspartate aminotransferase; ALT, alanine aminotransferase; AFP, α-fetoprotein*.

*p-values are obtained through comparison of the HV group, CHB group, and the CHC group*.

### Serum samples and protein labeling

Serum samples were immediately isolated from the clotted whole blood by centrifugation at 1,300 × g for 10 min at 4°C, which was immediately used or stored at −80°C. To normalize the differences between subjects and to tolerate individual variation, 5 μL of serum samples from all HV, CHB, and CHC patients were pooled, respectively. Then, 15, 22, and 23 individual serum samples of the HV, CHB and CHC patients were randomly selected for further validation. The protein concentration was determined by the Bradford protein assay. The pooled and individual serum samples were labeled with Cy3 fluorescent dye and purified using a Sephadex G-25 columns (Yu et al., 2012).

### Lectin microarrays

A lectin microarray was produced using 37 lectins [purchased from Vector Laboratories, Sigma-Aldrich (St Louis, MO) and Calbiochem (Billerica, MA)] with different binding preferences covering *N-* and *O-*linked glycan according to our previous protocol (Qin et al., 2013). Briefly, 37 lectins were dissolved in the manufacturer's recommended buffer containing 1 mmol/L of the appropriate monosaccharide at a concentration of 1 mg/mL and were spotted on the homemade epoxysilane-coated slides with Stealth micro-spotting pins (SMP-10B; TeleChem, Sunnyvale, CA) by a Capital smart microarrayer (CapitalBio, Beijing, China). Each lectin was spotted in triplicate per block with quadruplicate blocks on one slide. After immobilization, the slides were blocked with the blocking buffer containing 2% (w/v) BSA in 1× phosphate buffer saline (PBS) (0.01 mol/L phosphate buffer containing 0.15 mol/L NaCl, pH 7.4) for 1 h, then rinsed twice with 1× PBST (0.2% Tween 20 in 0.01 mol/L phosphate buffer containing 0.15 mol/L NaCl, pH 7.4) and 1× PBS. Then the appropriate amount of Cy3-labeled protein diluted in 0.5 mL of incubation buffer containing 2%(w/v) bovine serum albumin (BSA), 500 mM glycine, and 0.1% Tween-20 in 1× PBS was applied to the blocked lectin microarrays, and an incubation was performed in the chamber at 37°C for 3 h in a rotisserie oven set at 4 rpm. The slide was washed with 1× PBST twice for each for 5 min and washed once with 1× PBS for 5 min, and then dried by centrifugation at 600 rpm for 5 min.

### Microarray data processing and analysis

The microarrays were scanned at 70% photomultiplier tube and 100% laser power settings using a Genepix 4000B confocal scanner (Axon Instruments, Sunnyvale, CA). Genepix 3.0 software (Axon Instruments) was used to analyze the acquired images at 532 nm for Cy3 detection. The average background was subtracted and the values < average background ±2 standard deviation (SD) were removed from each data point. Median of the effective data points of each lectin was globally normalized to the sum of medians of all effective data points for each lectin in one block. Each sample was observed consistently by three repeated slides and the normalized medians of each lectin from nine repeated blocks were averaged and the SD was determined. The normalized data of each sample were compared with each other based upon fold-changes according to the following criteria: fold changes >2 or <0.5 indicated an up-regulation or a down-regulation, respectively. Differences between the two arbitrary data sets or multiple data sets were tested by Student's *t* test or one-way ANOVA to each lectin signal using SPSS v. 19. The original data was further analyzed by Expander 6.0 (http://acgt.cs.tau.ac.il/expander/) in order to perform a hierarchical clustering analysis.

### SDS-PAGE and lectin blotting analysis

The pooled sera of the HV, CHB, and CHC patients were analyzed by SDS-PAGE and subsequently lectin blotting (Blomme et al., 2009). For SDS-PAGE, samples were boiled for 5 min at 100°C mixed with 5 × loading buffer, and run on a 10% polyacrylamide resolving gel and a 5% stacking gel. Molecular mass standards (Thermo Scientific, Waltham, MA) were run with all gels. Some gels were then stained directly with alkaline silver. For lectin blotting, the proteins in gels were then transferred to a PVDF membrane (Immobilon-P; Millipore, Bedford, MA) with a semi-dry transfer unit (Hoefer Scientific, Holliston, MA) for 1.5 h at 24 mA. The membranes were washed twice with TTBS (150 mM NaCl, 10 mM Tris-HCl, 0.05% v/v Tween 20, pH 7.5) and then blocked for 1 h with Carbo-Free Blocking Solution (Vector, Burlingame, CA) at room temperature. On the basis of silver staining of the gels after transfer, it was evident that lower-molecular-mass proteins (<50 kDa) transferred more thoroughly to blots. Hence, if any bias was present in final results, it favored the detection of proteins smaller than 50 kDa during blotting with lectin. The membranes were then washed again and incubated with Cy5 (GE Healthcare) labeled lectins (2 μg/mL in Carbo-Free blocking solution) with gentle shaking overnight at 4°C in the dark. The membranes were washed twice each with TTBS for 10 min and scanned by red fluorescence channel (635 nm excitation/650LP emission) with the voltage of 800 PMT using a phosphorimager (Storm 840, Molecular Dynamics, Sunnyvale, CA).

### Selective isolation of glycoprotein fractions from sera by PHA-E+L-magnetic particle conjugates

The lectin-magnetic particle conjugate-based method was utilized to selectively isolate the glycoproteins according to our previous protocol (Yang et al., 2012, 2013). Briefly, 2 mg of the pooled serum proteins from the HV, CHB and CHC patients were diluted to 600 μL with the binding buffer (0.1 M Tris-HCl, 0.15 M NaCl, 1 mM CaCl_2_, 1 mM MgCl_2_, and 1 mM MnCl_2_, pH 7.4) supplemented with 6 μL proteinase inhibitor cocktail. The homemade PHA-E+L-magnetic particles were rinsed three times using the binding buffer, followed by incubation with the diluted sera at room temperature for 1 h under gentle shaking. After incubation, the unbound proteins were removed by washing three times with the washing buffer (the binding buffer supplemented with 0.1% Tween 20, pH 7.2) thoroughly. The glycoproteins bound to the PHA-E+L-magnetic particles were eluted with 500 μL of the elution buffer (8 M urea in 0.1 M NH_4_HCO_3_) at room temperature for 1 h under gentle shaking.

### Release and purification of *N*-glycans

The *N-*linked glycans were released from the isolated proteins with PNGase F according to our previous protocol with some modifications (Yang et al., 2012, 2013). The glycoproteins were concentrated and desalted by adding to a size-exclusion spin ultrafiltration (Amicon Ultra-0.5 10 K device, Millipore) with a molecular mass cutoff of 10 kDa. The obtained glycoproteins were denatured with 8 M urea, 10 mM DTT (Sigma-Aldrich), 10 mM IAM (Sigma-Aldrich), and 100 μL NH_4_HCO_3_ solutions (50 mM, pH 8.0). The ultrafiltration unit was centrifuged at 12,000 × g for 15 min followed by adding 10 μL of PNGase F (New England BioLabs, Ipswich, MA). Incubation was porformed at 37°C overnight to release the *N*-linked glycans from the glycoproteins. The reaction was stopped by incubating the mixture at 80°C for 5 min. The glycans were collected by centrifugation with 200 μL 40 mmol/L NH_4_HCO_3_. Then, the *N-*glycans were purified using Sepharose 4B (Sigma-Aldrich). Sepharose 4B in a microtube was washed with methanol/water (1:1, v/v) and 1-butanol/methanol/water (5:1:1, v/v) under centrifugation. Glycans were dissolved in 500 μL 1-butanol/methanol/water (5:1:1, v/v) and added to the microtube. The mixture was gently shaken for 45 min and washed three times with 1-butanol/methanol/water (5:1:1, v/v). *N-*glycans were eluted with methanol/water (1:1, v/v), and the eluent was collected and lyophilized.

### Analysis of *N*-glycan profiles

The glycan mixture was dissolved in 10 μL of 50% v/v methanol, and 1 μL were spotted directly on an MTP AnchorChip var/384 sample target and dried. Then an equal volume 20 mg/mL DHB in 50% v/v methanol solution was spotted to re-crystallize the glycans. The target was introduced in a mass spectrometer MALDI-TOF/TOF (UltrafleXtreme, Bruker Daltonics, Bremen, Germany). Ionization was performed in MS and MS/MS by irradiation of a nitrogen laser (337 nm) operating at 1 kHz. Data were acquired at a maximum accelerating potential of 25 kV in the positive and reflectron modes. Mass calibration was done using the peptide calibration standards 250 calibration points from Bruker Daltonics. A total of 1,500 laser shots per pixel (200 laser shots per position of a random walk within each pixel) were collected and the data were acquired using the Flex software suite (FlexControl3.3, Flex-Analysis 3.3). The data of *m/z* were analyzed and annotated by Glycoworkbench software (Ceroni et al., 2008). By combining the information from glycan structures recognized by a lectin and received from fragmentation of [M+H]^+^ and [M+Na]^+^ ions of the glycans, a complete structural characterization in terms of linkage, branching of glycosidic bonds of oligosaccharides were achieved.

## Results

### Glycopatterns of pooled sera from patients with CHB or CHC, and healthy volunteers

**Figure 1:**
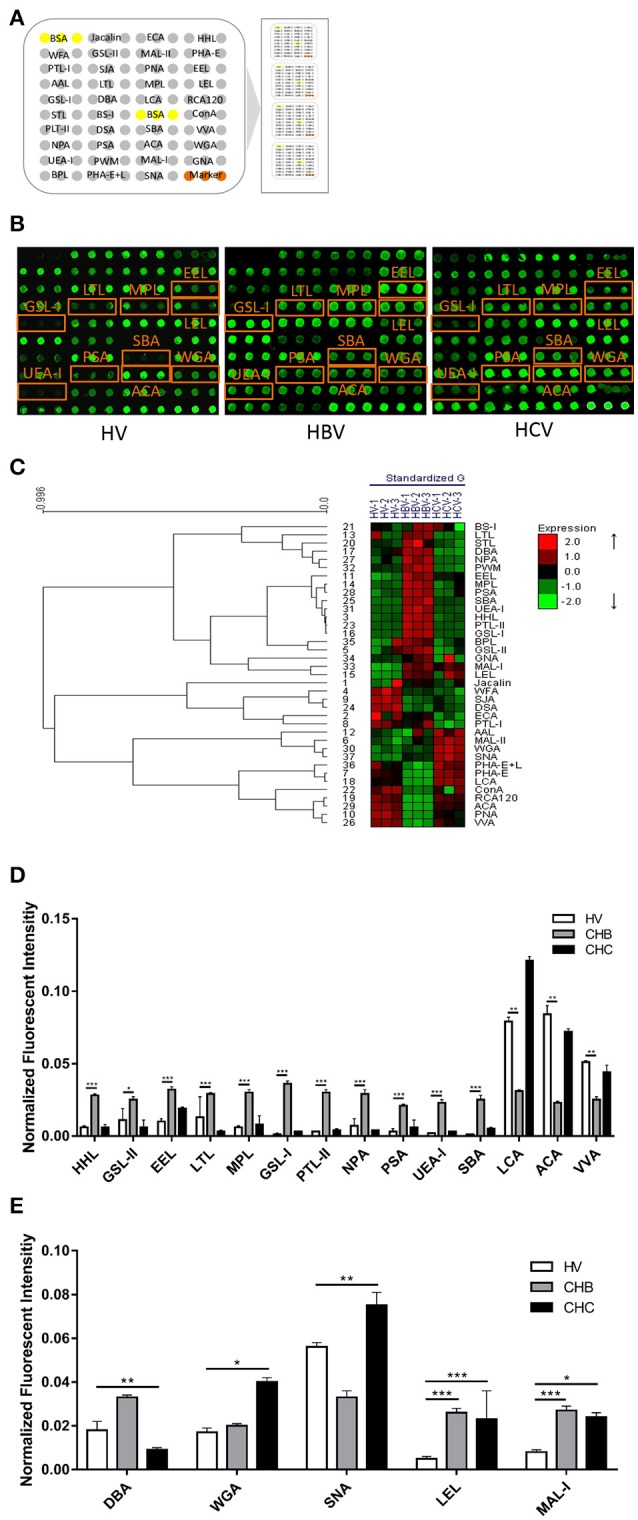
The glycopatterns of pooled sera glycoproteins from HV, CHB, and CHC patients using a lectin microarray. **(A)** The layout of the four area lectin microarray. Each lectin was spotted in triplicate per block, with quadruplicate blocks on one slide. Cy3-labeled BSA was spotted as a location marker and BSA as a negative control. **(B)** The profiles of Cy3-labeled pooled sera proteins from HVs and patients with CHB and CHC bound to the lectin microarrays. A portion of the slide with four replicates of the lectin microarrays was shown. The lectin microarrays revealed significant difference marked with orange frames. **(C)** Heat map and hierarchical clustering analysis of the 37 lectins for triplicate experiments. The samples were listed in columns, and the lectins were listed in rows. The color and intensity of each square indicated expression levels relative to the other data in the row. Red, high; green, low; black, medium. **(D)** Lectins showing significant alterations of NFIs exclusive to the CHB patients compared with HVs **(E)** Lectins showing significant alterations of NFIs exclusive to the CHC patients compared with HVs and lectins showing significant alterations of NFIs common in the CHB and CHC patients compared with HV according to one-way ANOVA (^*^p < 0.05, ^**^p < 0.01, and ^***^p < 0.001).

A lectin microarray format and the glycopatterns of Cy3-labeled pooled sera from the HV, CHB, and CHC patients bound to the lectin microarrays are shown in Figures 1A,B. The averaged normalized fluorescent intensities (NFIs) of each lectin from the HV, CHB, and CHC patients are summarized as the mean values ± SD in Table S1. The generated data from three biological replicates were imported into EXPANDER 6.0 to perform a hierarchical clustering analysis (Figure 1C). The NFIs of each lectin from the HV, CHB, and CHC patients were compared each other based on fold change in pairs (fold change >2.0 or <0.5 fold), with all *p*-values below 0.05, to evaluate whether the glycopatterns of serum proteins were altered between them (Table 2). The results showed that there were 19 lectins to exhibit significant difference associated with virus-infected hepatitis in serum glycopatterns compared with the HV. Among the total, there were 11 lectins (e.g., the High-Man and Manα1-3Man binder HHL, GlcNAc and agalactosylated tri/tetra antennary glycans binder GSL-II, and Galα1-3(Fucα1-2)Gal binder EEL) that exhibited significantly increased NFIs (all fold changes ≥ 2.19, *p* ≤ 0.05), and 3 lectins (the Fucα-1,6GlcNAc (core fucosylated) binder LCA, and Fucα-1,6GlcNAc binder ACA as well as GalNAcα-Ser/Thr(Tn) and GalNAcα1-3Gal binder VVA) exhibited significantly decreased NFIs (all fold changes ≤ 0.48, *p* ≤ 0.002) only in the CHB patients (Figure 1D). However, The GalNAcα1-3(Fucα1-2)Gal binder DBA exhibited significantly decreased NFIs (fold change = 0.47, *p* ≤ 0.004), the multivalent Sia and (GlcNAc)_*n*_ binder WGA, and Sia2-6Gal/GalNAc binder SNA exhibited significantly increased NFIs (all fold changes ≥ 2.00, *p* ≤ 0.05) only in the CHC patients. And the (GlcNAc)n and high mannose-type *N-*glycans binder LEL, and Galβ1-4/3GlcNAc binder MAL-I showed a coincidentally increasing trend in both CHC and CHB patients compared with the HV (all fold changes ≥ 2.97, *p* ≤ 0.058) (Figure 1E).

**Table 2:** Fold change of the pooled protein glycopatterns in sera from patients with CHB or CHC, and healthy volunteers (HV) based upon ratio of the NFIs of each lectin.

**Lectin**	**Specificity**	**Fold change(*p* value)^a^**		
		**CHB/HV**	**CHC/HV**	**CHC/CHB**
HHL	High-Mannose, Manα1-3Man, Manα1-6Man, Man5-GlcNAc2-Asn	5.01(<0.001)	–	0.21(<0.001)
GSL-II	GlcNAc and agalactosylated tri/tetra antennary glycans	2.35(0.04)	–	0.24(0.008)
EEL	Galα1-3(Fucα1-2)Gal (blood group B antigen)	3.33(0.001)	–	–
LTL	Fucα1-2Galβ1-4GlcNAc, Fucα1-3(Galβ1-4)GlcNAc, anti-H blood group specificity	2.19(0.05)	0.20(0.05)	0.09(0.019)
MPL	Galβ1-3GalNAc, GalNAc	4.99(<0.001)	–	0.26 (0.003)
LEL	(GlcNAc)n, high mannose-type N-glycans	5.62(0.034)	5.02(0.058)	–
GSL-I	αGalNAc, αGal, anti-A and B	24.59(<0.001)	–	0.07(0.007)
DBA	αGalNAc, Tn antigen, GalNAcα1-3((Fucα1-2))Gal (blood group A antigen)	–	0.47(0.004)	0.26(<0.001)
LCA	α-D-Man, Fucα-1,6GlcNAc, α-D-Glc	0.39(0.002)	–	3.93(0.002)
PTL-II	Gal, blood group H, T-antigen	9.67(<0.001)	–	0.12(<0.001)
SBA	α- or β-linked terminal GalNAc, (GalNAc)n, GalNAcα1-3Gal, blood-group A	16.58(<0.001)	3.15(0.040)	0.19(0.0012)
VVA	terminal GalNAc, GalNAcα-Ser/Thr(Tn), GalNAcα1-3Gal	0.48(0.002)	–	–
NPA	Galβ1-3GalNAcα-Ser/Thr(T-antigen)	3.97(<0.001)	–	0.14(<0.001)
PSA	Fucoseα-1,6GlcNAc(core fucose)	6.08(0.001)	–	0.27(0.002)
ACA	α-D-Man, Fucα-1,6GlcNAc, α-D-Glc	0.28(0.002)	–	3.08(0.002)
WGA	Multivalent Sia and (GlcNAc)n	–	2.32(0.013)	2.00(0.05)
UEA-I	Fucoseα1-2Galβ1-4Glc(NAc)	14.32(<0.001)	–	0.11(0.02)
MAL-I	Galβ1-4GlcNAc, Siaα2-3Gal, Galβ1-3GlcNAc, Siaα2-3	3.39(0.001)	2.97(0.005)	–
SNA	Sia2-6Galβ1-4Glc(NAc)	–	–	2.26(<0.001)

a *The NFIs of each lectin from CHB and CHC groups were compared with that from HV group based on their fold change (i.e., CHB/HV and CHC/HV)*.

—*, no significant difference*.

### Validation of different glycopatterns among patients with CHB or CHC, and healthy volunteers

**Figure 2:**
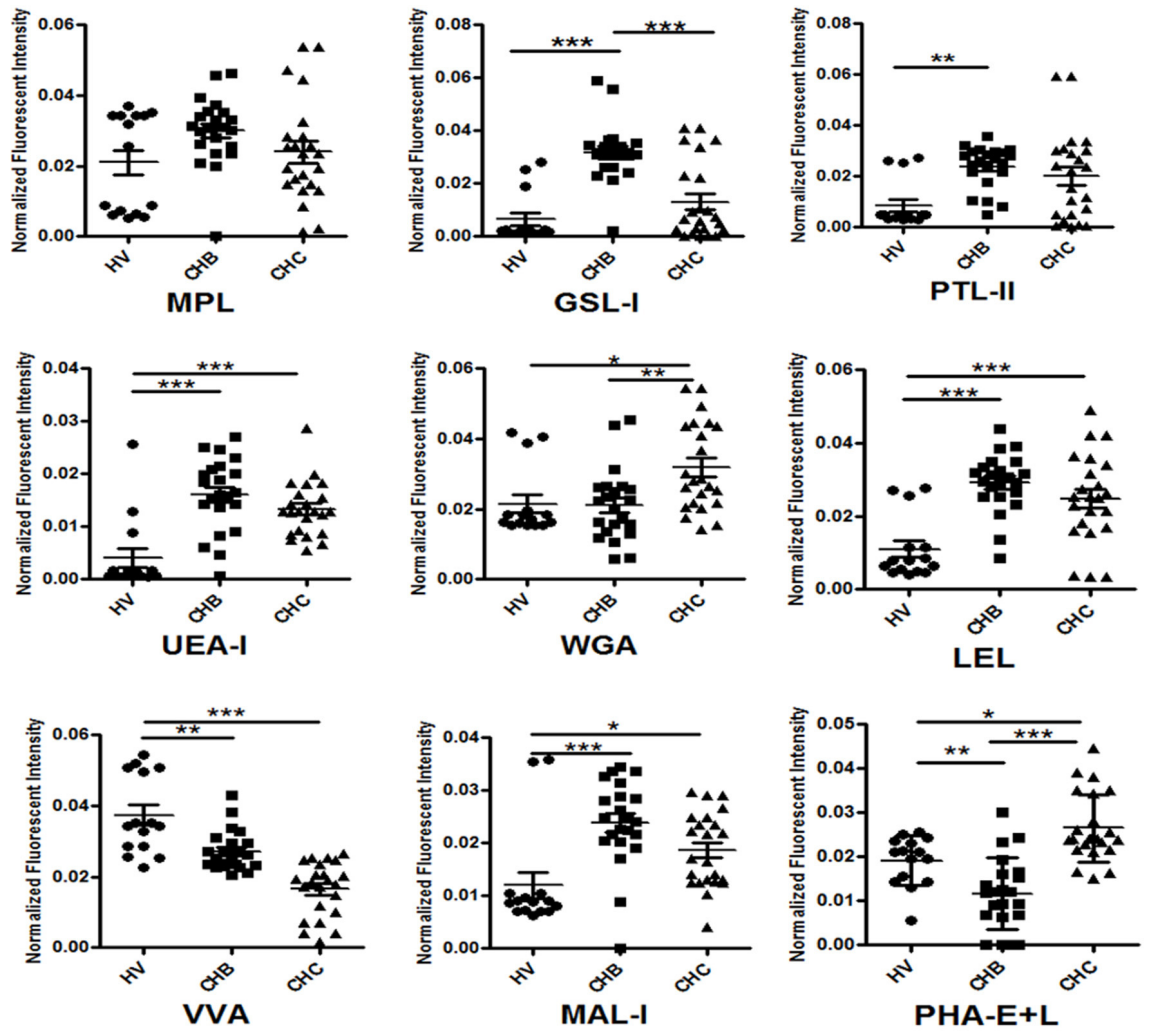
Individual glycopattern analysis in sera from HVs, CHB, and CHC patients. Scatterplots of the data were obtained with lectin microarrays. Significant differences between HVs, CHB, and CHC patients were analyzed by a non-parametric one-way ANOVA (^*^p < 0.05, ^**^p < 0.01, and ^***^p < 0.001).

To evaluate the reliability of the serum protein glycopatterns of the HV, CHB, and CHC patients from the results of the lectin microarrays, an additional cohort of 15 HV, 22 CHB, and 23 CHC patients was selected randomly and tested one by one using the same lectin microarrays. The results of individual analysis also demonstrated that there were eight lectins (MPL, GSL-I, PTL-II, UEA-I, WGA, LEL, VVA, and MAL-I) exhibited a high degree of consistency with the pooled samples of HV, CHB, and CHC patients. Besides, a bisecting GlcNAc, bi-antennary *N-*glycans, tri-, and tetra-antennary complex-type *N-*glycans binder PHA-E+L exhibited significantly decreased NFIs in the CHB compared with HV and CHC subjects (*p* < 0.01) (Figure 2). Notably, PHA-E+L were also associated with the decreased NFIs in the pooled serum samples of CHB compared with that of HV and CHC subjects (all fold changes ≤ 0.69, *p* < 0.001).

**Figure 3:**
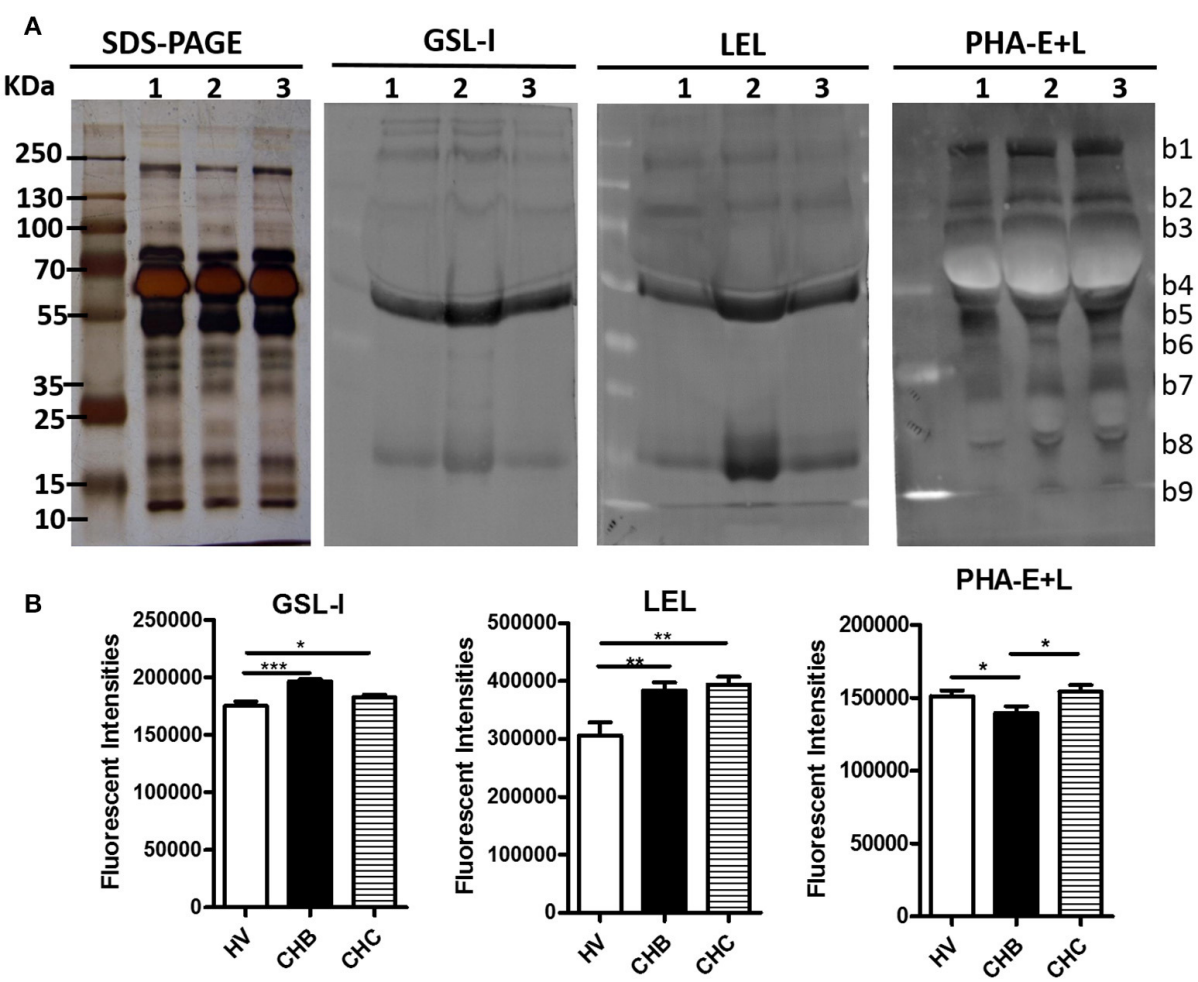
Validation of the differential expressions of the glycopatterns in the sera from CHB, CHC patients, and HV **(A)** The binding pattern of glycoproteins from the three groups of sera pooled samples using 3 lectins (GSL-I, LEL, and PHA-E+L). Lane 1, HV; lane 2, CHB patients; lane 3, CHC patients. Blot affinity results showed nine apparent bands belonging to different molecular weights, ranging from 10 to 250 kDa, which were marked as b1−b9, respectively. **(B)** Summed fluorescence intensities (read by Image J) along the distance from lane 1 to lane 9 for each lectin binding to sera.

Lectin blotting analyses (GSL-I, LEL, and PHA-E+L) were performed to investigate the difference of pooled serum glycopattern abundances between HV, CHB, and CHC patients (Figure 3A). The results of SDS-PAGE demonstrated that their serum protein bands were similar, the highest abundant bands with molecular weights (Mr) of 55–70 kDa is albumin. The result of the lectin blotting analysis showed the different apparent bands range from 10 to 250 kDa. The αGalNAc, αGal, and GalNAcα-Ser/Thr (Tn) binder GSL-I and the high mannose-type *N-*glycans binder LEL showed strong binding to three bands (b4, b5, b8) in the CHB patients than in the HV and CHC patients. On the contrary, PHA-E+L exhibited a weaker binding to mainly b5 band in the CHB patients compared with the HV and CHC patients. The sum fluorescence signal intensity of the bands for all subject groups was performed by Image J (Figure 3B). The results were coincident with the results from the lectin microarrays.

### Analysis of *N*-glycan profiles by MALDI-TOF/TOF-MS

**Figure 4:**
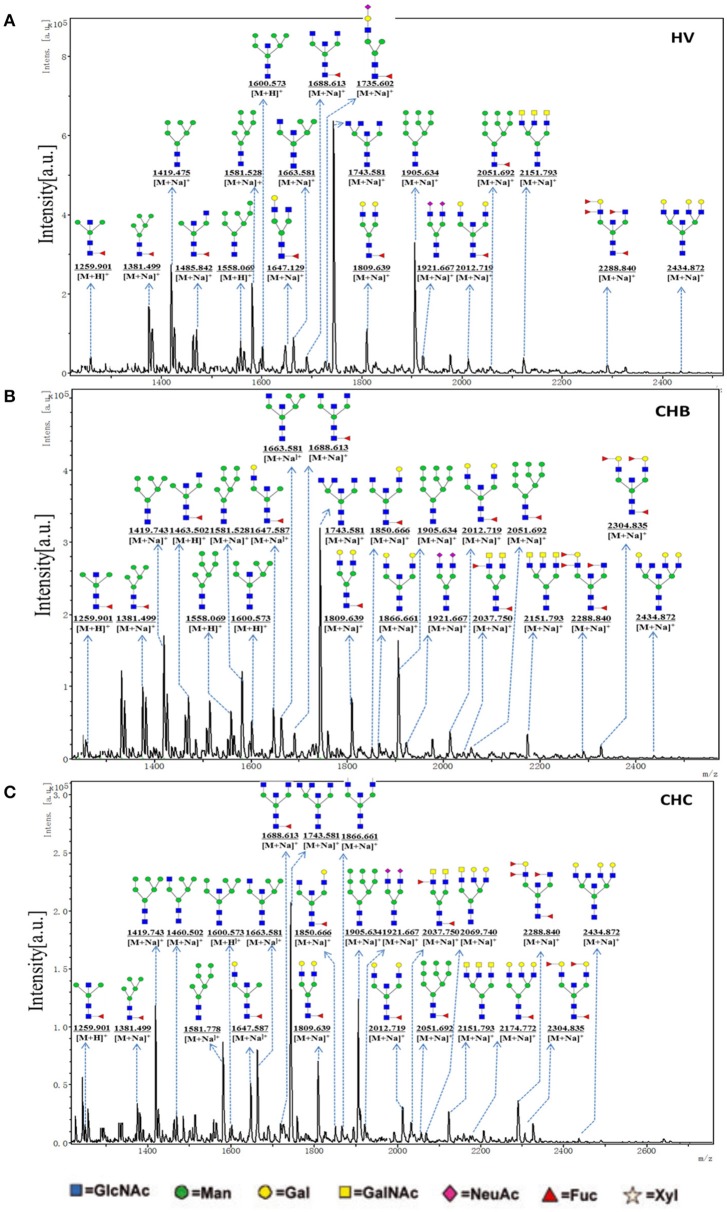
One MALDI-TOF-MS spectra of the PNGase F released *N*-linked glycan from the PHA-E+L affinity glycoproteins of the serum from HVs, CHB, and CHC patients. **(A)** Glycan spectra of HVs; **(B)** Glycan spectra of CHB patients; **(C)** Glycan spectra of CHC patients. Proposed structures and their m/z values are shown for each peak.

To obtain the bi-, tri-, and tetra-antennary complex-type *N-*glycan structures of glycoproteins in sera from the HV, CHB, and CHC patients, the glycoproteins were isolated using the PHA-E+L-magnetic particle conjugates, then *N*-glycans were released from the isolated glycoproteins by PNGase F, and characterized by MALDI-TOF/TOF-MS, respectively. Representative MS spectra of *N*-glycans with signal-to-noise ratios >6 were annotated using the GlycoWorkbench software. As a result, the MALDI-TOF/TOF-MS peaks of *N*-linked glycans from the isolated glycoproteins in sera of the HV, CHB, and CHC patients were shown in Figures 4A–C. There were 18 *N*-glycan peaks (e.g., *m/z* 1663.734, 1809.849, and 1921.809), 23 *N*-glycan peaks (e.g., *m/z* 1419.743, 1663.734, and 1743.581) and 26 *N*-glycan peaks (e.g., *m/z* 1866.661, 2012.908, and 2039.542) to be identified in HV, CHB, and CHC patients, respectively, and their proposed structures were listed in Table S2. Of these *N*-linked glycans, it was noticed that there was an overlap of 23 *N*-glycan peaks (e.g., *m/z* 1419.743, 1663.734, and 1743.581) between CHB, and CHC patients, 5 glycan peaks (e.g., *m/z* 1850.878, 1866.661, and 2037.750) were presented in virus-infected hepatitis patients compared with HV, 3 glycan peaks (e.g., 1460.659, 2069.740, and 2174.772) were observed only in CHC patients.

**Figure 5:**
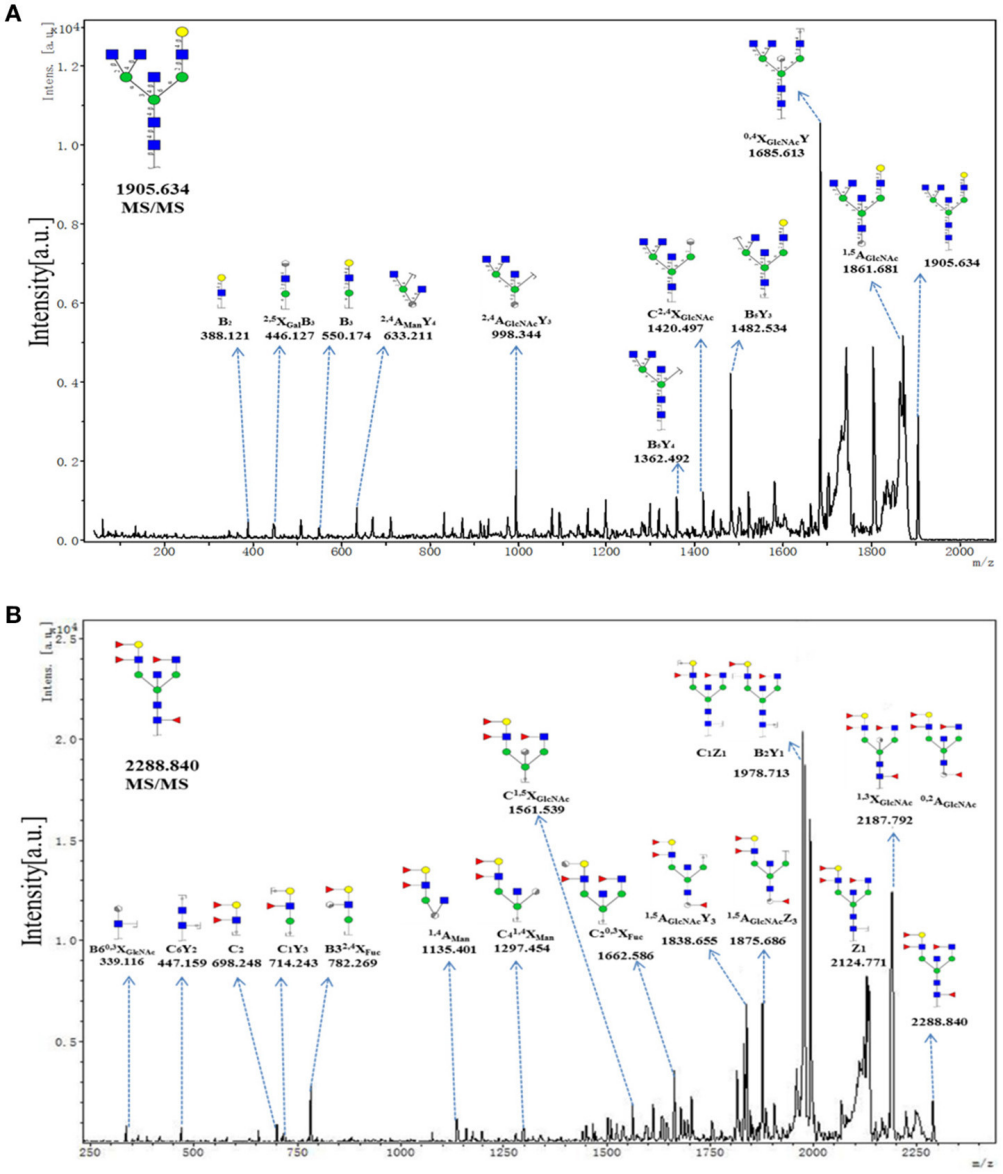
MS/MS analysis of N-glycan precursor ions in MS spectra. Precursor ions were subjected to MS/MS analysis to obtain cleavages, including glycosidic cleavages, and cross-ring cleavages. Structures of cleavage ions and *m/z*-values are shown in tandem mass spectra. Two major N-glycan peaks were indicated: **(A)** *m/z* 1905.634, and **(B)** 2288.840.

To provide insight into the substitution and branching pattern of the monosaccharide constituents, the majority of the glycan peaks observed in the CHC MS spectrum were assigned to tandem MS analysis. The MS/MS spectra of the precursor ions *m/z* 1905.634 and 2288.840 were illustrated in Figure 5. The results showed that the B-, C-, and Y-type cleavage that provided detailed sequence and branching information were the major fragment ion due to cleavage needed low energy. The cross-ring fragment ions that provide composition and linkage information were also detected. For instance, the fragment ions B_2_ (388.121), B_3_ (550.174), ^2, 4^A_GlcNAc_Y_3_ (998.344), and C^2, 4^X_GlcNAc_ (1420.497) in the *m/z* 1905.634 elucidated the condition of the triantennary *N-*glycans and the occurrence of the terminal β1,4Gal. Fragment ions C_2_ (692.248), C_1_Y_3_ (714.243), ${\text{B}}_{3}^{2,4}$X_Fuc_ (782.269), C^1, 5^X_GlcNAc_ (1561.539), and ^1, 5^A_GlcNAc_Y_3_ (1838.655) in the m/z 2288.840 indicated that the existence of both core and terminal fucose. In all, this method did not only demonstrate the overall difference in glycosylation, but also could reflect the differences between individual structures.

## Discussion

Alterations in the serum glycome result from proteins secreted or shed from the damaged tissue, and may also reflect the host response to the disease processes and cell status such as differentiation stage (Kuno et al., 2013). With regard to virus-infected chronic hepatitis, the presence of the aberrant glycans in sera is mainly due to the change of glycosyltransferase gene, which was caused due to virus gene or protein regulations. Another way is through B-lymphocytes autocrine and paracrine stimulation by the receptors of the inflammatory cytokines (Arnold et al., 2008), which lead to the abnormal glycosylation modifications in both core structures and terminal structures of glycans. In previous study, the HBV infection has been most extensively investigated in search of changes in glycosylation. Hyperfucosylation, increased branching and the presence of increased bisecting *N-*acetylglucosamine of glycans are clearly associated with different type of liver disease (Blomme et al., 2009). Anderson reported that glycosylation could serve as a discriminating factor between liver diseases using AGP as a model protein (Anderson et al., 2002). It is reported that the expression levels of core fucose and sialylated structures significantly increased in the CHC patients compared with the CHB patients. And, the expression levels of high-mannose and fucosylated structures increased in virus-infected hepatitis patients (Kang et al., 2004; Kuno et al., 2011). In this study, there were 19 lectins to exhibit significant difference associated with virus-infected hepatitis in pooled serum glycopatterns compared with the HV. Among the total, there were 11 lectins (HHL, GSL-II, EEL, LTL, MPL, GSL-I, PTL-II, VVA, NPA, PSA, and UEA-I) that exhibited significantly increased NFIs (all fold changes ≥ 2.19, *p* ≤ 0.05), and 3 lectins [the Fucα-1,6GlcNAc (core fucosylated) binder LCA, and Fucα-1,6GlcNAc binder ACA as well as GalNAcα-Ser/Thr(Tn) and GalNAcα1-3Gal binder VVA] exhibited significantly decreased NFIs (all fold changes ≤ 0.48, *p* ≤ 0.002) only in the CHB patients. However, The GalNAcα1-3(Fucα1-2)Gal binder DBA exhibited significantly decreased NFIs (fold change = 0.47, *p* ≤ 0.004), the multivalent Sia and (GlcNAc)_*n*_ binder WGA, and Sia2-6Gal/GalNAc binder SNA exhibited significantly increased NFIs (all fold changes ≥ 2.00, *p* ≤ 0.05) only in the CHC patients. And the (GlcNAc)_*n*_ and high mannose-type *N-*glycans binder LEL, and Galβ1-4/3GlcNAc binder MAL-I showed a coincidentally increasing trend in both CHC and CHB patients compared with the HV (all fold changes ≥ 2.97, *p* ≤ 0.058).

The individual analysis demonstrated that there were eight lectins (MPL, GSL-I, PTL-II, UEA-I, WGA, LEL, VVA, and MAL-I) exhibited a high degree of consistency with the pooled samples of HV, CHB, and CHC patients. Although, PHA-E+L were associated with the decreased NFIs in the pooled serum samples of CHB compared with that of HV and CHC subjects (all fold changes ≤ 0.69, *p* < 0.001), The results of individual analysis exhibited that PHA-E+L significantly decreased NFIs in the CHB compared with HV and CHC subjects (*p* < 0.01). To obtain the complex-type *N-*glycan structures of glycoproteins in sera from the HV, CHB, and CHC patients, the glycoproteins were isolated using the PHA-E+L-magnetic particle conjugates, then *N*-glycans were released from the isolated glycoproteins by PNGase F, and characterized by MALDI-TOF/TOF-MS. The representative MS spectra of *N*-linked glycans showed that there was an overlap of 23 *N*-glycan peaks (e.g., *m/z* 1419.743, 1663.734, and 1743.581) between CHB, and CHC patients, five glycan peaks (e.g., *m/z* 1850.878, 1866.661, and 2037.750) were presented in virus-infected hepatitis patients compared with HV, threeglycan peaks (1460.659, 2069.740, and 2174.772) were observed only in CHC patients. Using MALDI-TOF/TOF-MS, Yang et al. (Xiang et al., 2017) reported the complex-type glycan increased by 28.5% as well as tri- and tetra-antennary glycan increased by 30.9% in the HCV infected Huh7.5.1 cells compared with the non-infected Huh7.5.1. Also, their lectin microarray demonstrated that the NFIs of PHA-E and PHA-L has elevated in HCVcc-infected cells compared with control. Complex glycans at the cell surface play important roles; they serve as targets of microbes and viruses (Priyambada et al., 2017), in this study, the increase in multi-antennary and complex-type N-glycan may have correlation with the alteration of the glycosyltransferases. The detailed pathological mechanism should be researched, and the ROC curve including more clinical samples should be involved in the future study.

In conclusion, a new basic insight into the glycopatterns and complex-type *N-*glycans of glycoprotein in sera, and systematically compared their different or similar alterations between HBV and HCV-infected hepatitis patients. The present study provide useful information to find new biomarkers for distinguishing of CHB and CHC patients based on the precision alteration of their serum glycopatterns.

## Author contributions

XQ, YG, ZJ, and ZL designed the experiments. XQ, YG, YZ, JZ, XL, and ZZ performed the experiments. HD, JZ, and HY performed the data analysis. XQ, YG, HD, and ZL wrote the first draft and all authors contributed to review and revision and have seen and approved the final version.

### Conflict of interest statement

The authors declare that the research was conducted in the absence of any commercial or financial relationships that could be construed as a potential conflict of interest.

## References

[B1] Alter M. J. (2003). Epidemiology of hepatitis B in Europe and worldwide. J. Hepatol. 39(Suppl. 1), S64–S69. 10.1016/S0168-8278(03)00141-7 14708680

[B2] Jonas M. M. (2009). Hepatitis B and pregnancy: an underestimated issue. Liver Int. 29(Suppl. 1), 133–139. 10.1111/j.1478-3231.2008.01933.x 19207977

[B3] Arzumanyan A. Reis H. M. Feitelson M. A. (2013). Pathogenic mechanisms in HBV- and HCV-associated hepatocellular carcinoma. Nat. Rev. Cancer 13, 123–135. 10.1038/nrc3449 23344543

[B4] Sali S. Sharafi H. Alavian S. H. Alavian S. M. Etesam F. Salimi S.. (2015). Can serum level of HBsAg differentiate HBeAg-negative chronic hepatitis B from inactive carrier state? Diagn. Microbiol. Infect. Dis. 82, 114–119. 10.1016/j.diagmicrobio.2015.02.005 25863529

[B5] He Q. Y. Lau G. K. Zhou Y. Yuen S. T. Lin M. C. Kung H. F.. (2003). Serum biomarkers of hepatitis B virus infected liver inflammation: a proteomic study. Proteomics 3, 666–674. 10.1002/pmic.200300394 12748946

[B6] Chan H. L. (2002). Changing scene in hepatitis B serology interpretation. Hosp. Med. 63, 16–19. 10.12968/hosp.2002.63.1.1719 11828810

[B7] Li L. M. Hu Z. B. Zhou Z. X. Chen X. Liu F. Y. Zhang J. F.. (2010). Serum microRNA profiles serve as novel biomarkers for HBV infection and diagnosis of HBV-positive hepatocarcinoma. Cancer Res. 70, 9798–9807. 10.1158/0008-5472.CAN-10-1001 21098710

[B8] Hayes C. N. Chayama K. (2016). MicroRNAs as biomarkers for liver disease and hepatocellular carcinoma. Int. J. Mol. Sci. 17:280. 10.3390/ijms17030280 26927063 PMC4813144

[B9] Giray B. G. Emekdas G. Tezcan S. Ulger M. Serin M. S. Sezgin O.. (2014). Profiles of serum microRNAs; miR-125b-5p and miR223-3p serve as novel biomarkers for HBV-positive hepatocellular carcinoma. Mol. Biol. Rep. 41, 4513–4519. 10.1007/s11033-014-3322-3 24595450

[B10] Jimenez C. R. Verheul H. M. (2014). Mass spectrometry-based proteomics: from cancer biology to protein biomarkers, drug targets, and clinical applications. Am. Soc. Clin. Oncol. Educ. Book e504–e510. 10.14694/EdBook_AM.2014.34.e504 24857147

[B11] Zhang L. Huang Y. Lian M. Fan Z. Tian Y. Wang Y.. (2017). Metabolic profiling of hepatitis B virus-related hepatocellular carcinoma with diverse differentiation grades. Oncol. Lett. 13, 1204–1210. 10.3892/ol.2017.5596 28454235 PMC5403281

[B12] Xia B. Zhang W. Li X. Jiang R. Harper T. Liu R.. (2013). Serum N-glycan and O-glycan analysis by mass spectrometry for diagnosis of congenital disorders of glycosylation. Anal. Biochem. 442, 178–185. 10.1016/j.ab.2013.07.037 23928051

[B13] Kuno A. Uchiyama N. Koseki-Kuno S. Ebe Y. Takashima S. Yamada M.. (2005). Evanescent-field fluorescence-assisted lectin microarray: a new strategy for glycan profiling. Nat. Methods 2, 851–856. 10.1038/nmeth803 16278656

[B14] Blomme B. Van Steenkiste C. Callewaert N. Van Vlierberghe H. (2009). Alteration of protein glycosylation in liver diseases. J. Hepatol. 50, 592–603. 10.1016/j.jhep.2008.12.010 19157620

[B15] Dwek R. A. Butters T. D. Platt F. M. Zitzmann N. (2002). Targeting glycosylation as a therapeutic approach. Nat. Rev. Drug Discov. 1, 65–75. 10.1038/nrd708 12119611

[B16] Liu X. E. Desmyter L. Gao C. F. Laroy W. Dewaele S. Vanhooren V.. (2007). N-glycomic changes in hepatocellular carcinoma patients with liver cirrhosis induced by hepatitis B virus. Hepatology 46, 1426–1435. 10.1002/hep.21855 17683101

[B17] Arnold J. N. Saldova R. Hamid U. M. Rudd P. M. (2008). Evaluation of the serum N-linked glycome for the diagnosis of cancer and chronic inflammation. Proteomics 8, 3284–3293. 10.1002/pmic.200800163 18646009

[B18] Kang S. K. Chung T. W. Lee J. Y. Lee Y. C. Morton R. E. Kim C. H. (2004). The hepatitis B virus X protein inhibits secretion of apolipoprotein B by enhancing the expression of N-acetylglucosaminyltransferase III. J. Biol. Chem. 279, 28106–28112. 10.1074/jbc.M403176200 15123606

[B19] Tsai W. L. Chung R. T. (2010). Viral hepatocarcinogenesis. Oncogene 29, 2309–2324. 10.1038/onc.2010.36 20228847 PMC3148694

[B20] Honda M. Yamashita T. Ueda T. Takatori H. Nishino R. Kaneko S. (2006). Different signaling pathways in the livers of patients with chronic hepatitis B or chronic hepatitis C. Hepatology 44, 1122–1138. 10.1002/hep.21383 17058214

[B21] Gornik O. Lauc G. (2008). Glycosylation of serum proteins in inflammatory diseases. Dis. Markers 25, 267–278. 10.1155/2008/493289 19126970 PMC3827815

[B22] Yu H. Zhu M. Qin Y. Zhong Y. Yan H. Wang Q.. (2012). Analysis of glycan-related genes expression and glycan profiles in mice with liver fibrosis. J. Proteome Res. 11, 5277–5285. 10.1021/pr300484j 23043565

[B23] Qin Y. Zhong Y. Zhu M. Dang L. Yu H. Chen Z.. (2013). Age- and sex-associated differences in the glycopatterns of human salivary glycoproteins and their roles against influenza A virus. J. Proteome Res. 12, 2742–2754. 10.1021/pr400096w 23590532

[B24] Yang G. Cui T. Chen Q. Ma T. Li Z. (2012). Isolation and identification of native membrane glycoproteins from living cell by concanavalin A-magnetic particle conjugates. Anal. Biochem. 421, 339–341. 10.1016/j.ab.2011.10.033 22079135

[B25] Yang G. Cui T. Wang Y. Sun S. Ma T. Wang T.. (2013). Selective isolation and analysis of glycoprotein fractions and their glycomes from hepatocellular carcinoma sera. Proteomics 13, 1481–1498. 10.1002/pmic.201200259 23436760

[B26] Ceroni A. Maass K. Geyer H. Geyer R. Dell A. Haslam S. M. (2008). GlycoWorkbench: a tool for the computer-assisted annotation of mass spectra of glycans. J. Proteome Res. 7, 1650–1659. 10.1021/pr7008252 18311910

[B27] Kuno A. Ikehara Y. Tanaka Y. Ito K. Matsuda A. Sekiya S.. (2013). A serum sweet-doughnut protein facilitates fibrosis evaluation and therapy assessment in patients with viral hepatitis. Sci. Rep. 3:1065. 10.1038/srep01065 23323209 PMC3545220

[B28] Anderson N. Pollacchi A. Hayes P. Therapondos G. Newsome P. Boyter A.. (2002). A preliminary evaluation of the differences in the glycosylation of alpha-1-acid glycoprotein between individual liver diseases. Biomed. Chromatogr. 16, 365–372. 10.1002/bmc.167 12228891

[B29] Kuno A. Ikehara Y. Tanaka Y. Angata T. Unno S. Sogabe M.. (2011). Multilectin assay for detecting fibrosis-specific glyco-alteration by means of lectin microarray. Clin. Chem. 57, 48–56. 10.1373/clinchem.2010.151340 21047982

[B30] Xiang T. Yang G. Liu X. Zhou Y. Fu Z. Lu F.. (2017). Alteration of N-glycan expression profile and glycan pattern of glycoproteins in human hepatoma cells after HCV infection. Biochim. Biophys. Acta 1861(5 Pt A), 1036–1045. 10.1016/j.bbagen.2017.02.014 28229927

[B31] Priyambada S. A. Misaki R. Okamoto T. Okamoto Y. Ohashi T. Ueda K. (2017). Cell surface N-glycan alteration in HepAD38 cell lines expressing Hepatitis B virus. Virus Res. 238, 101–119. 10.1016/j.virusres.2017.06.003 28645725

