# Supplementary material for: Comparative Analysis for Glycopatterns and Complex-Type N-Glycans of Glycoprotein in Sera from Chronic Hepatitis B- and C-Infected Patients
Source: Front Physiol. 2017 Aug 21;8:596. doi: 10.3389/fphys.2017.00596 (PMC5566988; doi:10.3389/fphys.2017.00596)
Supplement: Supplementary file 1 [file Table1.DOCX]

Supplementary Material

Comparative Analysis for Glycopatterns and Complex-type N-Glycans of Glycoprotein in Sera from Chronic Hepatitis B - and C -Infected Patients

Xinmin Qin^†^, Yonghong Guo^†^, Haoqi Du^†^, Yaogang Zhong, Jiaxu Zhang, Xuetian Li, Hanjie Yu, Zhiwei Zhang, Zhansheng Jia*, Zheng Li*

†These authors contributed equally to this work.

*** Correspondence:**

Zheng Li, Laboratory for Functional Glycomics, College of Life Sciences, Northwest University, Xi'an, China. E-mail: zhengli@nwu.edu.cn

Zhansheng Jia, Department of Infectious Diseases and Center for Liver Diseases, Tangdu Hospital, the Fourth Military Medical University, Xi’an, China. E-mail: jiazsh@fmmu.edu.cn

# Supplementary Table

**Supplementary Table S1.** Glycopattern in sera from patients with CHB or CHC, and healthy volunteers (HV) by the lectin microarray analysis based on data of 37 lectins giving significant signal.

| Lectin | Specificity | NFI±SD^a^ | | |
| --- | --- | --- | --- | --- |
|  |  | HV | CHB | CHC |
| Jacalin | Galβ1-3GalNAcα-Ser/Thr(T), GalNAcα-Ser/Thr(Tn), GlcNAcβ1-3-GalNAcα-Ser/Thr(Core3), sialyl-T(ST) | 0.023±0.008 | 0.024±0.001 | 0.022±0.002 |
| ECA | Galβ-1,4GlcNAc (type II), Galβ1-3GlcNAc (type I) | 0.030±0.011 | 0.025±0.002 | 0.015±0.004 |
| HHL | High-Mannose, Manα1-3Man, Manα1-6Man, Man5-GlcNAc2-Asn | 0.006±0.001 | 0.028±0.001 | 0.006±0.002 |
| WFA | terminating in GalNAcα/β1-3/6Gal | 0.041±0.004 | 0.027±0.001 | 0.023±0.004 |
| GSL-II | GlcNAc and agalactosylated tri/tetra antennary glycans | 0.011±0.008 | 0.025±0.002 | 0.006±0.005 |
| MAL-II | Siaα2-3Galβ1-4Glc(NAc)/Glc, Siaα2-3Gal, Siaα2-3, Siaα2-3GalNAc | 0.030±0.001 | 0.020±0.003 | 0.029±0.001 |
| PHA-E | Bisecting GlcNAc, biantennary complex-type N-glycan with outer Gal | 0.037±0.001 | 0.026±0.000 | 0.045±0.002 |
| PTL-I | GalNAc, GalNAcα-1,3Gal, GalNAcα-1,3Galβ-1,3/4Glc | 0.021±0.002 | 0.020±0.004 | 0.012±0.001 |
| SJA | Terminal in GalNAc and Gal, anti-A and anti-B human blood group | 0.045±0.002 | 0.025±0.002 | 0.027±0.004 |
| PNA | Galβ1-3GalNAcα-Ser/Thr(T) | 0.062±0.004 | 0.033±0.001 | 0.049±0.003 |
| EEL | Galα1-3(Fucα1-2)Gal (blood group B antigen) | 0.010±0.002 | 0.032±0.002 | 0.019±0.001 |
| AAL | Fucα1-6 GlcNAc(core fucose), Fucα1-3(Galβ1-4)GlcNAc | 0.017±0.002 | 0.019±0.006 | 0.026±0.003 |
| LTL | Fucα1-2Galβ1-4GlcNAc, Fucα1-3(Galβ1-4)GlcNAc, anti-H blood group specificity | 0.013±0.014 | 0.029±0.001 | 0.003±0.001 |
| MPL | Galβ1-3GalNAc, GalNAc | 0.006±0.001 | 0.030±0.002 | 0.008±0.006 |
| LEL | (GlcNAc)n, high mannose-type N-glycans | 0.005±0.001 | 0.026±0.002 | 0.023±0.013 |
| GSL-I | αGalNAc, αGal, anti-A and B | 0.001±0.001 | 0.036±0.002 | 0.003±0.000 |
| DBA | αGalNAc, Tn antigen, GalNAcα1-3((Fucα1-2))Gal (blood group A antigen) | 0.018±0.004 | 0.033±0.001 | 0.009±0.001 |
| LCA | α-D-Man, Fucα-1,6GlcNAc, α-D-Glc | 0.079±0.003 | 0.031±0.001 | 0.121±0.003 |
| RCA_120_ | β-Gal, Galβ-1,4GlcNAc (type II), Galβ1-3GlcNAc (type I) | 0.067±0.005 | 0.024±0.001 | 0.066±0.004 |
| STL | trimers and tetramers of GlcNAc, core (GlcNAc) of N-glycan, oligosaccharide containing GlcNAc and MurNAc | 0.044±0.002 | 0.064±0.009 | 0.043±0.004 |
| BS-I | α-Gal, α-GalNAc, Galα-1,3Gal, Galα-1,6Glc | 0.016±0.001 | 0.020±0.002 | 0.017±0.003 |
| ConA | High-Mannose, Manα1-6(Manα1-3)Man, terminal GlcNAc | 0.069±0.009 | 0.026±0.002 | 0.047±0.032 |
| PTL-II | Gal, blood group H , T-antigen | 0.003±0.000 | 0.030±0.002 | 0.004±0.001 |
| DSA | β-D-GlcNAc, (GlcNAcβ1-4)n, Galβ1-4GlcNAc | 0.050±0.001 | 0.036±0.002 | 0.039±0.002 |
| SBA | aα- or β-linked terminal GalNAc, (GalNAc)n, GalNAcα1-3Gal, blood-group A | 0.001±0.000 | 0.025±0.003 | 0.005±0.001 |
| VVA | terminal GalNAc, GalNAcα-Ser/Thr(Tn), GalNAcα1-3Gal | 0.051±0.001 | 0.025±0.002 | 0.044±0.005 |
| NPA | High-Mannose, Manα1-6Man | 0.007±0.005 | 0.029±0.003 | 0.004±0.000 |
| PSA | α-D-Man, Fucα-1,6GlcNAc, α-D-Glc | 0.003±0.002 | 0.021±0.001 | 0.006±0.005 |
| ACA | Galβ1-3GalNAcα-Ser/Thr (T antigen), sialyl-T(ST) | 0.084±0.006 | 0.023±0.001 | 0.072±0.002 |
| WGA | Multivalent Sia and (GlcNAc)n | 0.017±0.002 | 0.020±0.001 | 0.040±0.002 |
| UEA-I | Fucα1-2Galβ1-4Glc(NAc) | 0.002±0.000 | 0.023±0.002 | 0.003±0.000 |
| PWM | Branched (LacNAc)n | 0.013±0.001 | 0.026±0.001 | 0.010±0.001 |
| MAL-I | Galβ-1,4GlcNAc, Siaα2-3Gal, Galβ1-3GlcNAc, Siaα2-3 | 0.008±0.001 | 0.027±0.002 | 0.024±0.002 |
| GNA | High-Mannose, Manα1-3Man | 0.017±0.000 | 0.022±0.002 | 0.019±0.007 |
| BPL | Galβ1-3GalNAc, Terminal GalNAc | 0.011±0.011 | 0.021±0.002 | 0.010±0.004 |
| PHA-  E+L | Bisecting GlcNAc, bi-antennary N-glycans, tri- and tetra-antennary complex-type N-glycan | 0.022±0.003 | 0.015±0.001 | 0.026±0.002 |
| SNA | Sia2-6Gal/GalNAc | 0.056±0.002 | 0.033±0.003 | 0.075±0.006 |

a) Normalized fluorescent intensities (NFIs) obtained for 12 repeated blocks in 4 repeated slides were averaged and its SD was counted.

**Supplementary Table S2.** The proposed N-linked glycan structures for signal peaks detected in the present study.

| No. | Experimental  m/z | Calculated  m/z | Glycan structure | Serum Samples | | | Charge |
| --- | --- | --- | --- | --- | --- | --- | --- |
|  |  |  |  | HVs | CHB | CHC |  |
| 1 | 1259.901 | 1260.472 | 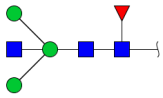 | ＋ | ＋ | ＋ | [M+H]^+^ |
| 2 | 1381.967 | 1381.499 | 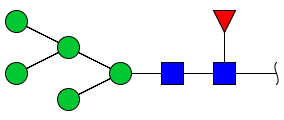 | ＋ | ＋ | ＋ | [M+H]^+^ |
|  | 1403.579 | 1403.481 |  |  |  |  | [M+Na]^+^ |
| 3 | 1419.743 | 1419.475 | 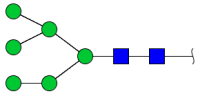 | ＋ | ＋ | ＋ | [M+Na]^+^ |
| 4 | 1460.659 | 1460.502 | 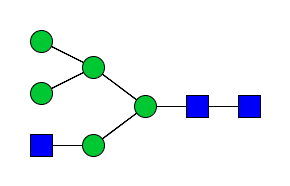 | ND | ND | ＋ | [M+Na]^+^ |
| 5 | 1463.940 | 1463.552 | 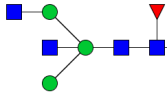 | ＋ | ＋ | ＋ | [M+H]^+^ |
|  | 1485.537 | 1485.842 |  |  |  |  | [M+Na]^+^ |
| 6 | 1558.053 | 1558.069 | 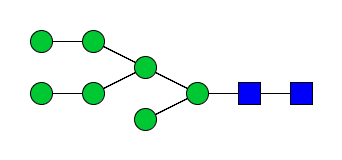 | ＋ | ＋ | ＋ | [M+H]^+^ |
|  | 1581.688 | 1581.528 |  |  |  |  | [M+Na]^+^ |
| 7 | 1601.101 | 1600.573 | 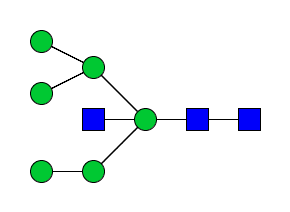 | ＋ | ＋ | ＋ | [M+H]^+^ |
| 8 | 1647.129 | 1647.587 | 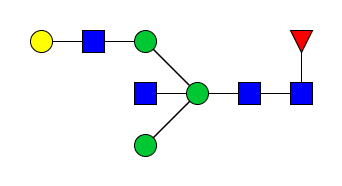 | ＋ | ＋ | ＋ | [M+Na]^+^ |
| 9 | 1663.734 | 1663.581 | 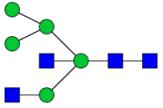 | ＋ | ＋ | ＋ | [M+Na]^+^ |
| 10 | 1690.148 | 1688.613 | 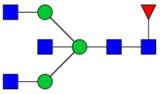 | ＋ | ＋ | ＋ | [M+Na]^+^ |
| 11 | 1734.426 | 1735.602 | 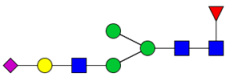 | ＋ | ＋ | ＋ | [M+Na]^+^ |
| 12 | 1745.634 | 1743.581 | 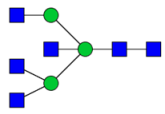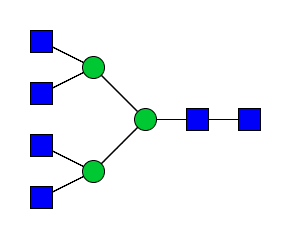 | ＋ | ＋ | ＋ | [M+Na]^+^ |
| 13 | 1809.849 | 1809.639 | 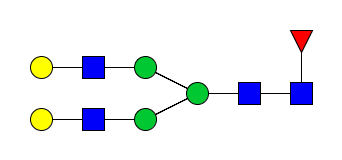 | ＋ | ＋ | ＋ | [M+Na]^+^ |
| 14 | 1850.878 | 1850.666 | 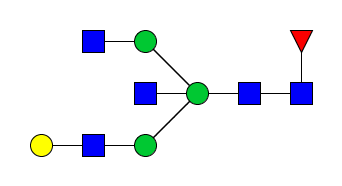 | ND | ＋ | ＋ | [M+Na]^+^ |
| 15 | 1866.661 | 1866.465 | 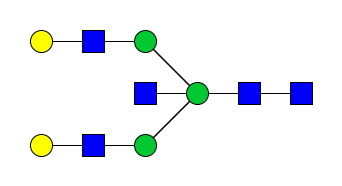 | ND | ＋ | ＋ | [M+Na]^+^ |
| 16 | 1905.829 | 1905.634 | 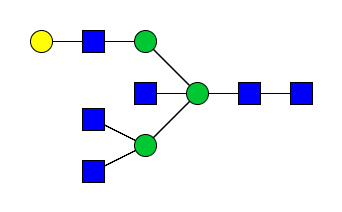 | ＋ | ＋ | ＋ | [M+Na]^+^ |
| 17 | 1921.809 | 1921.667 | 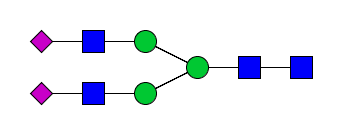 | ＋ | ＋ | ＋ | [M+Na]+ |
| 18 | 2012.908 | 2012.719 | 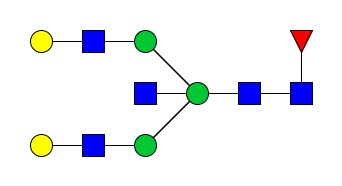 | ＋ | ＋ | ＋ | [M+Na]^+^ |
| 19 | 2039.542 | 2037.750 | 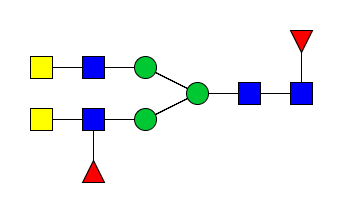 | ND | ＋ | ＋ | [M+Na]^+^ |
| 20 | 2050.205 | 2051.692 | 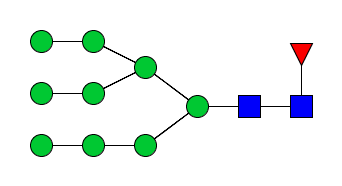 | ＋ | ＋ | ＋ | [M+Na]^+^ |
| 21 | 2068.368 | 2069.740 | 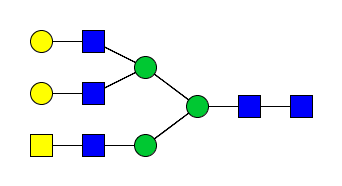 | ND | ND | ＋ | [M+Na]^+^ |
| 22 | 2151.959 | 2151.793 | 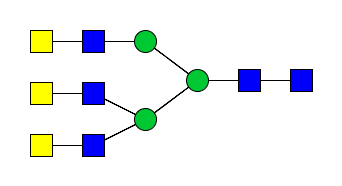 | ND | + | ＋ | [M+Na]^+^ |
| 23 | 2175.124 | 2174.772 | 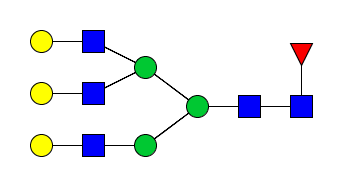 | ND | ND | ＋ | [M+Na]^+^ |
| 24 | 2289.831 | 2288.840 | 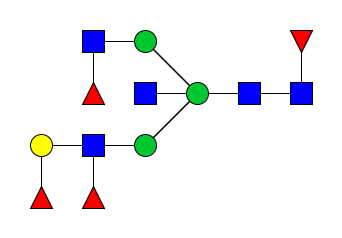 | ＋ | ＋ | ＋ | [M+Na]^+^ |
| 25 | 2305.936 | 2304.835 | 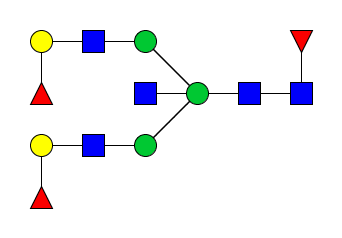 | ND | + | ＋ | [M+Na]^+^ |
| 26 | 2436.238 | 2434.872 | 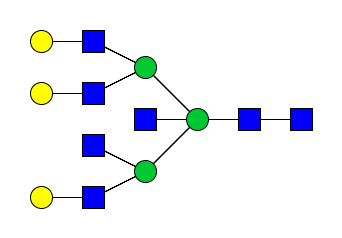 | ＋ | ＋ | ＋ | [M+Na]^+^ |

a) Monosaccharides are reprensented according to MS-tools from EUROCarbDB. GlcNAc, blue square; GalNAc, yellow square; Man, green circle; Gal, yellow circle; Fuc, red triangle; Neu5Ac, purple diamond.

b) +, detected in the samples; ND, not detected in the samples.
